# Ethnopharmacological Study of *Garrya laurifolia* and Its Antidiabetic Effect in Rats

**DOI:** 10.3390/plants13223235

**Published:** 2024-11-18

**Authors:** María Mirian Estévez-Carmona, Saudy Saret Pablo-Pérez, Jesús Eduardo Almanza-Cruz, María Estela Meléndez-Camargo, Daniel Arrieta-Baez, José Melesio Cristóbal-Luna, Margarita Franco-Colín

**Affiliations:** 1 Laboratorio de Farmacología y Toxicología Renal y Hepática, Departamento de Farmacia, Escuela Nacional de Ciencias Biológicas, Instituto Politécnico Nacional, Mexico City 07738, Mexico; mmestevez@ipn.mx (M.M.E.-C.); jalmancru@gmail.com (J.E.A.-C.); 2 Centro de Nanociencias y Micro y Nanotecnologías, Instituto Politécnico Nacional, Mexico City 07738, Mexico; darrieta@ipn.mx; 3 Laboratorio de Toxicología Preclínica, Departamento de Fisiología, Escuela Nacional de Ciencias Biológicas, Instituto Politécnico Nacional, Mexico City 07738, Mexico; josmcl@hotmail.com; 4 Laboratorio de Metabolismo I, Departamento de Fisiología, Escuela Nacional de Ciencias Biológicas, Instituto Politécnico Nacional, Mexico City 07738, Mexico; mfrancoc@ipn.mx

**Keywords:** *Garrya laurifolia*, ethnopharmacology, ethnobotanist, streptozotocin, antidiabetic, hypoglycemic, antihyperglycemic, herb–drug interaction

## Abstract

This study aimed to scientifically validate the traditional use of *Garrya laurifolia* (Gl) leaves as an antidiabetic agent attributed to a community in Mexico. The descriptive ethnobotanical study was conducted in San Miguel Tecpan, a municipality of Jilotzingo, State of Mexico, Mexico, where a structured questionnaire was applied to 44 inhabitants. *In vivo* studies evaluated the acute oral toxicity of Gl leaves in murine and the effects of a leaf infusion on glycemia in normoglycemic and diabetic rats; in addition, the interaction between Gl and metformin (Met) was also evaluated. The in vitro antioxidant activity of Gl was determined. The phytochemical screening and quantification of phenolic and flavonoid content of Gl leaves were performed. Gl had a high relative frequency of citation (0.68) among respondents. Gl had a low acute toxicity risk with LD_50_ > 5000 mg/kg. The extract had no hypoglycemic effect in normoglycemic rats, but it did have hypoglycemic and antihyperglycemic effects (250 and 500 mg/kg) in diabetic rats. The interaction between Gl (500 mg/kg) + Met (300 mg/kg) resulted in antidiabetic synergism. Gl showed strong antioxidant activity (93.1 ± 0.4%). Phytochemical screening revealed the presence of alkaloids, flavonoids, and some other phenolic compounds. The total phenol content was 77.9 ± 0.6 mg EQ/g and 87.7 ± 0.7 mg EAG/g, and the flavonoids content was 5.32 ± 0.2 mg EQ/g. UHPLC-MS/MS analysis identified chlorogenic acid, rutin, aucubin, luteolin 7-*O*-neohesperoside, and myricitrin. The findings support the potential use of Gl as a safe and effective antidiabetic agent.

## 1. Introduction

Diabetes mellitus (DM) is a pandemic non-transmissible chronic disease that globally affects approximately 537 million people between 20 and 79 years of age [1]. It is characterized by a high level of glucose in the blood due to the pancreas not secreting enough insulin or the insulin secreted not being used correctly by the body [2], with several related micro- and macro-vessel comorbidities such as irreversible damage to the kidneys, nerves, eyes, etc. [3].

People with diabetes need to maintain healthy habits as well as pharmacological treatment to control blood glucose, in addition to other medications to reduce the risk of complications [2]. It should be noted that the effectiveness of drugs, as well as their safety and access to patients, is limited. According to the World Health Organization (WHO), 75–80% of the world population uses traditional and complementary medicine, mainly herbal remedies, as a primary health source [4], and there is an increased demand for herbal drugs and depleting natural plant resources.

Using the ethnobotanical approach, data on the human use of plants and their inter-relationships have been studied, coming from ancient knowledge transmitted orally between generations [5]. However, much information is still missing on species with medicinal uses.

Mexico has had an increased percentage of patients with DM in the last few years, and social security is not enough to treat or palliate the symptoms of this disease or its medical complications [6]. Patients with DM search for accessible medical and alternative treatments, such as the consumption of medicinal plants, since the country has an incredible biodiversity of plants and a centuries-old culture of medicinal herbalism [5].

**Figure 1:**
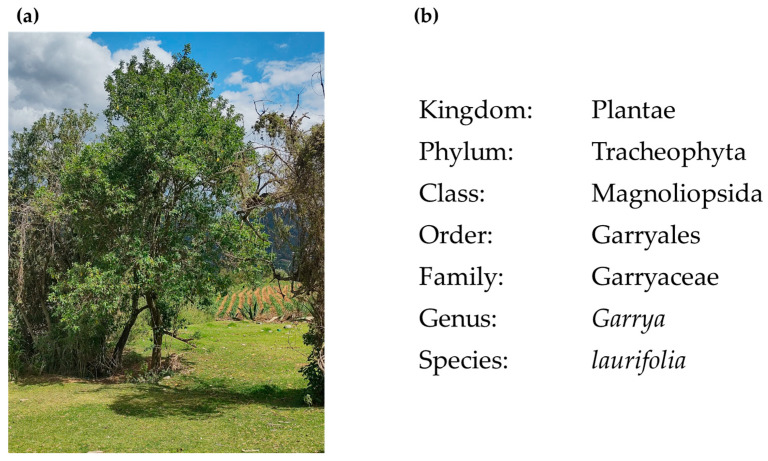
*Garrya laurifolia*. (**a**) Presence in the San Miguel Tecpan region, municipality of Jilotzingo, State of Mexico, Mexico (May 2023). (**b**) Taxonomical classification [9].

**Figure 2:**
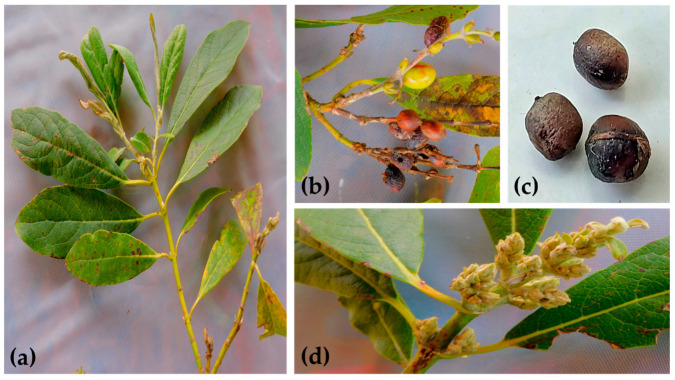
Structures of *G. laurifolia*. (**a**) Stem and leaves, (**b**) fruits, (**c**) seeds, and (**d**) inflorescence.

*Garrya laurifolia* Hartw. ex Benth, commonly named “chichicaule”, “bitter wood”, or “zapotillo”, is a native perennial shrub (Figure 1 and Figure 2) distributed widely in Mexico and Central America [7]. Previously, it has been reported that the leaves of *G. laurifolia* (Gl) contain the alkaloid garrifoline, which has mild hypotensive action in anesthetized cats, as well as mild antihistamine and anticholinergic activity [8]. Traditionally, the inhabitants of the State of Mexico, Mexico use an infusion of Gl leaves to reduce glycemia in conjunction with their medical treatment; however, no scientific reports support this activity or its interaction with hypoglycemic drugs and its toxicity.

This work aims to provide scientific support for the empirical use of Gl as an antidiabetic agent in a model of induced DM in rats.

## 2. Results

### 2.1. Descriptive Ethnobotanical Study

A representative sample of 17 men (39%) and 27 women (61%) who had knowledge of medicinal plants was surveyed. The age range of informants was from 29 to 89 years old, and the age group with the most significant representation was between 41 and 60 years (50%), followed by those between 61 and 89 years (36%), and finally, those under 40 years (14%).

In the area studied, evidence was obtained that the population empirically uses popular plants in the region to treat diabetes. Table S1 summarizes the information on medicinal plants orally consumed to treat DM by the population of San Miguel Tecpac, Jilotzingo, State of Mexico, Mexico. Gl was the third most cited species among the people surveyed, with a relative frequency of citation (RFC) of 0.68.

- Ethnomedicinal information of Gl

Most people recognize “chichicaule” as a native esthetic and medicinal shrub of the region. According to traditional knowledge, the medicinal uses that people attribute to Gl include hypoglycemic effects (83%), reducing the blood concentration of cholesterol and triglycerides (7%), tranquilizing and improving mood (2%), and finally, reducing inflammation and stomach pain (2%).

To achieve the empirical hypoglycemic effect, people mainly consume an infusion (83%) of five wild-collected Gl leaves in one liter of water during the day. According to this information, the protocol was developed to give scientific support to the widespread use of this plant.

### 2.2. In Vivo Studies of Gl

#### 2.2.1. Acute Toxicity Study

The infusion of Gl administered orally at different doses in female mice and rats did not produce mortality or any sign of toxicity during the entire test period. The lethal dose 50 (LD_50_) of infusion was more than 5000 mg/kg b.w. in NIH mice and female Wistar rats.

#### 2.2.2. Pharmacologic Study

- Effect on glycemia in the experimental DM model

**Figure 3:**
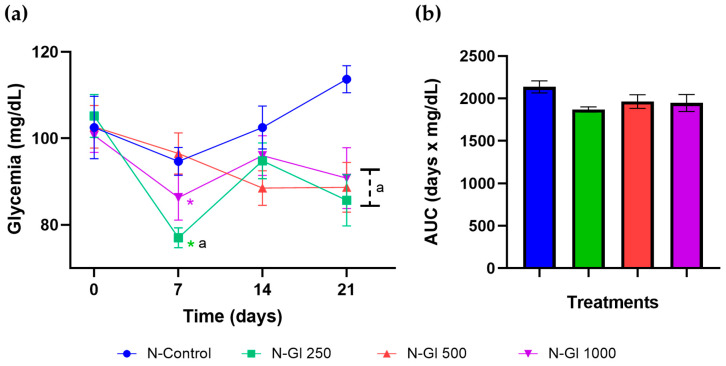
Effect of infusion of *G. laurifolia* leaves on glycemia in normoglycemic rats. (**a**) Time course of glycemia. Mean ± SEM (n = 6). Two-way repeated-measures ANOVA, S-N-K, *p* < 0.05, ^a^ compared to N-control, * compared to the measurement at the beginning of treatment. (**b**) AUC of the glycemic curve. One-way ANOVA, S-N-K, *p* > 0.05.

The infusion of Gl at 250 mg/kg decreased glycemia in normoglycemic rats on day 7; however, all doses of extract produced hypoglycemia compared to the N-control group on day 21 of oral treatment (Figure 3a). To monitor the effectiveness of glycemic control during the treatment period, the area under the curve (AUC) was calculated; no infusion dose decreased glycemia (Figure 3b).

**Figure 4:**
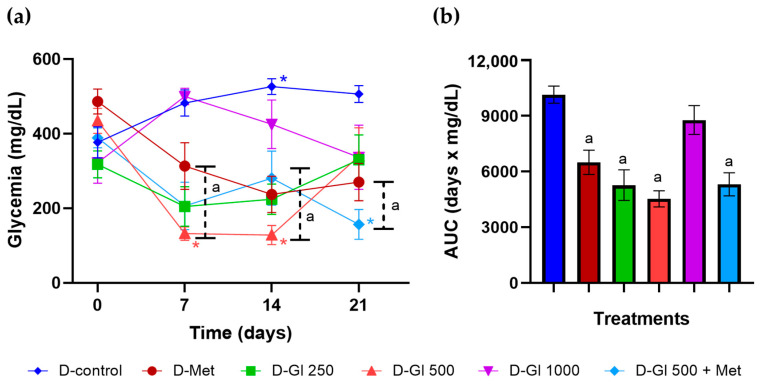
Effect of infusion of *G. laurifolia* leaves on glycemia in diabetic rats. (**a**) Time course of glycemia. Mean ± SEM (n = 6). Two-way repeated-measures ANOVA, S-N-K, *p* < 0.05, ^a^ compared to D-control, * compared to the measurement at the beginning of treatment. (**b**) AUC of the glycemic curve. One-way ANOVA, S-N-K, *p* < 0.05, ^a^ compared to D-control.

The hypoglycemic activity of Gl was observed on days 7 and 14 in diabetic rats treated with an infusion at doses of 250 and 500 mg/kg, while metformin (300 mg/kg) and their interaction (D-Gl 500 + Met) showed a hypoglycemic effect from day 7 and until the end of the treatment compared to the D-control (Figure 4a). The overall impact of the treatments on glycemia in diabetic rats showed that metformin, the plant infusion (250 and 500 mg/kg), and the herb–drug interaction are hypoglycemic (Figure 4b).

- Effect on Glucose Tolerance Test (GTT)

**Figure 5:**
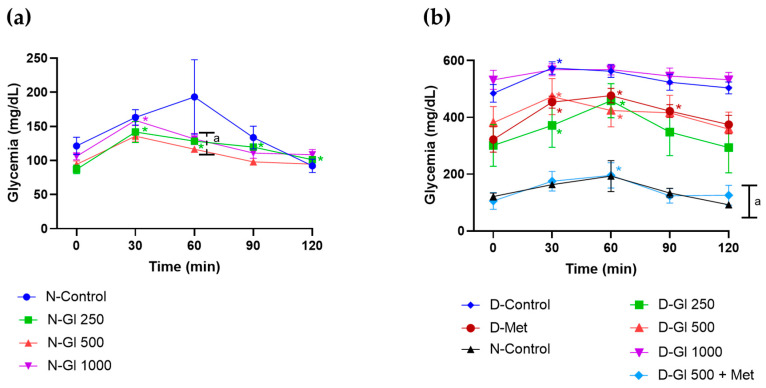
Effect of infusion of *G. laurifolia* leaves on glucose tolerance test. (**a**) In normoglycemic rats. Mean ± SEM (n = 6). Two-way repeated-measures ANOVA, S-N-K, *p* < 0.05, ^a^ compared to N-control, * compared to baseline measurement before glucose bolus administration (2 g/kg). (**b**) In diabetic rats. Mean ± SEM (n = 6). Two-way repeated-measures ANOVA, S-N-K, *p* < 0.05, ^a^ compared to the rest of the groups, * compared to baseline measurement before glucose bolus administration (2 g/kg).

The antihyperglycemic effect of Gl leaf infusion on GTT was evident at minute 60 after glucose loading in normoglycemic animals (Figure 5a). Interestingly, diabetic animals treated with the interaction (DE-Gl 500 + Met) had glycemia like the normoglycemic group (N-control) during the entire GTT time (Figure 5b), demonstrating that the interaction is more effective than the single administration of metformin (D-Met) or the infusion (D-Gl 500) separately.

### 2.3. Antioxidant Potential In Vitro

The infusion of Gl leaves had antioxidant potential of 93.1 ± 0.4%, while the antioxidant potency of quercetin was 92.9 ± 0.3%.

### 2.4. Phytochemical Studies of the Infusion of Gl Leaves

- Preliminary phytochemical screening

The identified specialized metabolites in the infusion of dry leaves were alkaloids, flavonoids (flavanones, xanthones, and flavones), and some other phenolic compounds, such as chlorogenic acid.

- Total phenolic and flavonoid

The infusion of dried leaves of Gl contained the following total phenol content: 77.9 ± 0.6 mg EQ/g of leaf and 87.7 ± 0.7 mg EAG/g of leaf. Regarding the flavonoid content, 5.32 ± 0.2 mg EQ/g of a leaf was found, representing approximately 6.4% of the total phenolic content.

- Ultra-High-Performance Liquid Chromatography Mass Spectrometry (UHPLC-MS/MS) analysis

The infusion of the dry leaves of Gl was analyzed by UHPLC-MS/MS analysis in negative mode, and the compounds identified are reported in Table 1. The chromatogram obtained can be viewed in the Supplementary Materials, Figure S1.

The main compounds identified in the analysis were [4-methyl-7-(1-naphthylmethoxy)-2-oxo-2H-chromen-3-yl]acetic acid (30.0%), chlorogenic acid isomer (25.0%), rutin (14.0%), ixoside (9.5%), aucubin (2.1%), luteolin 7-*O*-neohesperidoside (2.0%), and myricitrin (0.4%).

## 3. Discussion

In the present work, it has been observed that the oldest interviewed people living in a rural area of San Miguel Tecpan have more knowledge and experience about the use and benefits of medicinal plants than younger people, in concordance with similar reports [10]. The preservation of traditional knowledge about the medicinal properties of plants, recorded by ethnobotanical studies, is relevant for new generations because plants have been a source of effective drugs against several diseases for centuries and, recently, people have been interested in the significant consumption of herbal remedies rather than industrial medicine [4]. On the other hand, this kind of study had limitations such as the availability and memory of informants, the lack of participation, and distrust towards interviewers, which may have affected the data collection.

Although Gl is already traditionally used by people, it is essential to know at what doses the plant causes toxic or fatal effects in a population. This study demonstrated that the LD_50_ of the infusion of Gl leaves is more than 5000 mg/kg in murine animals, indicating a relatively low risk of toxicity [11]. However, subacute and chronic toxicity studies are necessary.

The only reported medicinal uses of Gl are to treat dysentery by Aztec healers [12] and as an antidiarrheal agent [13], so it is necessary to provide information about the current medicinal uses of this plant in Mexico. Interestingly, in the study area, an infusion of Gl leaves was identified as a traditional herbal remedy to treat DM; however, there are no previous reports about the anti-diabetic properties of this plant or the *Garrya* genus.

To evaluate the effect of Gl on glycemia in DM, the streptozotocin (STZ)-induced diabetes mellitus model was used; STZ is a drug chemically related to other nitrosureas used in cancer chemotherapy, capable of initiating an autoimmune process that results in damage and the death of pancreatic β cells, depending on the dose, through different mechanisms including DNA alkylation, the depletion of cellular NAD^+^ levels and therefore energy deprivation, increased oxidative stress, and increasing nitric oxide production, with the onset of clinical diabetes disease in 2 to 4 days. This model causes hyperglycemia, hypoinsulinemia, polyphagia, polyuria, and polydipsia accompanied by weight loss in adult rats within three days of induction [14,15]; most of these symptoms were observed in the diabetic-induced animals of this experiment. Likewise, it is essential to emphasize that in vivo animal models may not fully replicate human metabolic responses, thus limiting clinical extrapolation.

To better understand the antidiabetic potential of the plant, ensuring that its use is safe and effective in populations with and without diabetes, before evaluating the hypoglycemic effect of the infusion in diabetic rats, it was also evaluated in normoglycemic rats. According to the results of this work, it is possible to consume a Gl infusion in conditions of normoglycemia for three weeks without risk of severe hypoglycemia; however, the plant does have an antihyperglycemic effect after a glucose load, perhaps because it can decrease the glucose absorption in the gastrointestinal tract, which reduces the amount of glucose entering the circulation, as has been reported for other herbal extracts [16,17]. More studies are necessary to clarify these hypotheses.

Gl produces a hypoglycemic effect (250 and 500 mg/kg), not dose-dependent, in diabetic rats, which may be due to the suppression of gluconeogenesis, as reported in the case of berberine, an alkaloid from *Berberis vulgaris* [18]. The fact that the plant did not show antihyperglycemic or hypoglycemic effects at high doses (1000 mg/kg) can be explained by hormesis, a biphasic dose–response relationship characterized by stimulation at low doses and inhibition at high doses, which has been described in some substances such as metformin [19] and constituents of hypoglycemic plants such as ginseng [20], among others.

Many diabetic patients are known to use herbal medicines in addition to their conventional treatments, which may present both a potential benefit and risk to the effective treatment of their disease. There needs to be more data on herb–drug interactions [21]. This study shows that the interaction between the consumption of the infusion of Gl leaves (500 mg/kg) and metformin (300 mg/kg) produces synergism in the hypoglycemic effect.

This is the first time that the quantification of total phenols and flavonoids and the presence of tannins in the leaves of Gl have been reported, and their antioxidant activity in vitro has been determined. It should be noted that there are also no data to compare Gl with other species of its genus. The phenolic compounds, including flavonoids and other phenolic compounds, such as chlorogenic acid, act as antioxidants by reacting with various free radicals. The mechanism of antioxidant actions involves the transference of hydrogen atoms, single electrons, and electrons by the sequential loss of protons or transition metal chelation [22]. Compounds from Gl leaves induce high antioxidant activity, which is essential in treating DM, first, by preventing the dysfunction of pancreatic β cells, if any remain viable, caused by ROS accumulation resulting from prolonged hyperglycemia [23], and second, by preventing micro- and macrovascular complications associated with oxidative stress [24]. Although it cannot predict the antioxidant effect in vivo, this is a helpful basic test for determining the reducing capacity in vitro.

In the UHPLC-MS analysis of the infusion of Gl dried leaves, several compounds were found that contribute to the antihyperglycemic and hypoglycemic activity observed in the in vivo model of DM. For example, phenolic compounds like chlorogenic acid and its isomer, with 1.8% and 25% abundance in Gl, respectively, and the flavonoid rutin or vitamin P, with 13% abundance in Gl, have reported antioxidant [25] and hypoglycemic activity related to its content [26,27]. Proposed mechanisms for the antihyperglycemic effect of rutin include a decrease in carbohydrate absorption from the small intestine, an inhibition of tissue gluconeogenesis, an increase in tissue glucose uptake, the stimulation of insulin secretion from ß cells, and the protection of Langerhans islets against degeneration. Rutin also decreases the formation of sorbitol, reactive oxygen species, advanced glycation end-product precursors, and inflammatory cytokines [28]. Interestingly, the analysis also found aucubin (2.1%), an iridoid glycoside from the Garryaceae family, with antidiabetic and retinoprotective activities, among other valuable activities in the complications of DM [29]. Luteolin 7-*O*-neohesperidoside (4.2%) has been described as an antioxidant [30]. Myricitrin, a flavonoid with potent antioxidant and antidiabetic activity [31], was also found in the infusion in a glycoside form (myricetin-3-*O*-rhamnoside) in 4.6% abundance.

The presence of alkaloids in Gl agrees with Djerassi et al. (1955), who previously isolated two diterpenoid alkaloids, garrifoline and cuauchichicine, from the bark of a shrub from the State of Mexico, Mexico [13,32]; our work team is carrying out the corresponding analyzes to identify and quantify the alkaloids in the leaves by UHPLC-MS/MS.

## 4. Materials and Methods

### 4.1. Descriptive Ethnobotanical Study

- Description of the study area

The research was conducted in San Miguel Tecpan, a rural community in the municipality of Jilotzingo, State of Mexico, Mexico (19°31′54.740″ N, 99°23′54.719″ W), with an altitude of 2831 m (Figure S2). The total population is 1611, of which, 796 are women and 815 are men. The total number of inhabited homes is 418. Its primary economic activity is raising sheep. Furthermore, the community does not have a public clinic or health center, but it does have access to private medical services [33].

- Field work

The ethnobotanical study was conducted in March 2023. The study area was visited to hold conversations with inhabitants of the rural community and inform them of the objectives of this study. A snowball sampling method was used to identify critical informants for ethnobotanical interviews. Initial visits to local homes and public spaces led to recommendations from community members, allowing us to select participants recognized for their expertise in traditional plant knowledge. This approach ensured access to in-depth insights into local medicinal practices [34].

Semi-structured interviews were conducted, and the plants used to treat DM were investigated. Then, a structured questionnaire was applied to determine the knowledge about Gl, its common names, the method of obtainment, the part of the plant used, the quantity used, the preparation method, the frequency of use, and whether it is consumed together with standard medication (Figure S3) [35]. The relative frequency of citation (RFC) of the species was calculated according to Equation (1).

RFC = FC/N(1)

where FC is the frequency of citation and N is the number of informants who participated in the survey. The most used species in this index would obtain the highest citation frequency among the community members [36]. A search was carried out in the literature to determine if there is scientific evidence of the antidiabetic activity of the reported plants. Additionally, information was obtained on sociocultural aspects such as the sex and age of the interviewees.

### 4.2. Collection and Identification of Botanical Material

Leaves, flowers, stems, seeds, and fruits of the plant, popularly known by the community as “chichicaule”, and photographic material of the plant in its native environment were collected with the permission of local authorities in May 2023. A specimen of the plant was identified by the expert PhD María de la Luz Arreguín-Sánchez, with the help of the specialized literature [37], and compared to a voucher specimen deposited in the National Herbarium of Mexico (MEXU), Vascular Plants, under number 643718. The leaves of Gl were dried at room temperature, crushed, and stored in a dry place until use.

### 4.3. Reagents and Drugs

Folin–Ciocalteu phenol reagent (FC), aluminum chloride (AlCl_3_), STZ, and 1,1-Diphenyl-2-picrylhydrazyl (DPPH•) were acquired from Sigma-Aldrich (St. Louis, MO, USA). Quercetin was procured from Fluka (Buchs, Switzerland). Other reagents and solvents were obtained from local sources and were of analytical grade or better.

### 4.4. Preparation of Infusion of G. laurifolia Leaves

The aqueous extract of Gl leaves was infused using dried and crushed leaves of plant material. Briefly, water of appropriate quality was boiled in a beaker and removed from the heat source; a quantity of leaves was immediately added and mixed with the water, and the baker was covered; the infusion was allowed to stand for 5 min before performing any assay. Daily, a fresh leaf infusion was prepared for each phytochemical test and oral administration to the experimental animals.

### 4.5. In Vivo Studies of Gl

- Animal care and housing

Female nulliparous and non-pregnant Wistar rats (200 ± 20 g b.w.) and NIH mice (30 ± 5 g b.w.) were used for the acute toxicity study. Meanwhile, adult female nulliparous and non-pregnant Wistar rats (200 ± 20 g b.w.) were used for the pharmacologic study. They were housed and maintained in an animal house at room temperature (25 ± 2 °C) and 50 ± 5% relative humidity, with light/dark cycles of 12 × 12 h. The rodents’ standard diet and water were available *ad libitum*. The use and care of the experimental animals were carried out following national and international guidelines on the welfare of experimental animals [38,39] and with institutional ethical committee approval (CEI-ENCB-004/2015).

#### 4.5.1. Acute Toxicity Study

The objective of this study was to determine the lethal dose 50 (LD_50_, dose capable of causing death in 50% of the animals) of the Gl leaf infusion after a single administration in mice and confirm the result in rats, since the rat is the most sensitive species, and the one used in the pharmacological study.

Female mice and rats were used after fasting overnight with water *ad libitum*. On the day of the experiment, twenty mice were randomized into five groups: (1) the control (vehicle, water 1 mL/kg b.w.) and (2–5) those who received different doses of leaf infusions of Gl at 625, 1250, 2500, and 5000 mg/kg b.w., respectively. Additionally, eight rats were randomized into two groups: (1) the control (vehicle, water 10 mL/kg b.w.) and (2) those who received a plant infusion at a dose of 5000 mg/kg b.w. In all groups, administration was carried out once *per os*.

All animals were observed during the next three hours post-administration and daily during the ulterior 14 days to register any change in skin and fur; eyes and mucous membranes; respiratory, circulatory, autonomic, and central nervous systems; and somatic motor activity and behavior patterns, as well as observations of tremors, convulsions, salivation, diarrhea, lethargy, sleep, coma, and death, according to the Organization for Economic Cooperation and Development (OECD) guideline 423 (Figure S4) [11].

#### 4.5.2. Pharmacologic Study

The objective of the study was to equalize the conditions of the traditional oral consumption of the medicinal plant Gl in an animal model of DM (Figure S5).

- Induction of experimental DM in rats

Rats were randomized into two groups. The first group, the control normoglycemic group (N), received 0.1 M citrate buffer (pH 4.5) intraperitoneally (1 mL/kg b.w., IP), and the second group, the diabetic (D) induction group, was administered freshly diluted STZ (65 mg/kg b.w., IP) in citrate buffer only once.

Then, 72 h post-STZ administration, glycemia was determined in 12 h fasted animals (access to water *ad libitum*) by measuring their glucose in a blood sample obtained from the tails using a glucometer, One-touch^®^ Select Plus Flex™ (Flextronics Industrial Co., Ltd., Shenzhen, China). Animals with glycemia greater than 150 mg/dL were considered diabetic [15].

- Effect of Gl on glycemia

Immediately upon measuring basal glycemia, normoglycemic rats were divided into four groups: (1) the N-control (vehicle, water 1 mL/kg b.w.) and (2–4) infusion of Gl leaves (250, 500, and 1000 mg/kg b.w. and N-Gl 250, N-Gl 500, and N-Gl 1000, respectively). Diabetic rats were randomized into six groups: (1) D-control (vehicle, water 1 mL/kg b.w.), (2) metformin (D-Met, 300 mg/kg b.w.), (3–5) infusion of Gl leaves (250, 500, and 1000 mg/kg b.w. and D-Gl 250, D-Gl 500, and D-Gl 1000, respectively), and (6) drug interaction (D-Gl 500 + Met). All treatments were administered *per os* daily for 21 days.

On days 7, 14, and 21 of the treatments, glucose levels in all 12 h fasted animals were determined in tail-blood samples by glucose test stripes in the glucometer [40]. At the end of the experiment, the animals were sacrificed.

- Effect of Gl on Glucose Tolerance Test (GTT)

The GGT was performed on day 18 of the treatments. The basal glycemia of all animals fasting for 12 h was measured, and a glucose load was immediately administered *per os* (2 g/kg b.w.). Subsequently, the glucose concentration was measured again at 30, 60, 90, and 120 min post-glucose loading [41].

### 4.6. Antioxidant In Vitro Activity of Gl

The ability of the extract to scavenge DPPH• free radicals was estimated [42]. An aliquot of 1 mL of the infusion of Gl leaves was mixed with 1 mL of a freshly prepared DPPH• methanol solution (63.4 μM). A control was measured in the same way, except that the extract was replaced by methanol. After 30 min of incubation in darkness and ambient temperature, the absorbance was recorded at 514 nm. The antioxidant activity of the extract was expressed as a percentage according to Equation (2), as follows:

% antioxidant activity = [1 − A_sample_/A_control_)] × 100 (2)

where A is the absorbance. A quercetin solution at the same concentration as the sample was used as a positive control. The values are presented as the means ± SEM (standard error of mean) of triplicate analyses in all reported quantifications.

### 4.7. Phytochemical Studies of Gl

- Preliminary phytochemical screening

Standard qualitative phytochemical color and precipitation tests detected the specialized metabolites present in the infusion of Gl leaves. Various chemical reactions were used to demonstrate the presence of alkaloids, flavonoids, phenolic compounds, coumarins, saponins, quinones, cardiotonic glycosides, sesquiterpenlactones, and cyanogenetic glycosides [43].

- Total phenolic content

The total phenolic content was assessed by using FC reagent according to the method previously reported with slight modifications [44]. An aliquot (20 μL) of appropriately diluted infusion or standard was added into test tubes, and then, 100 μL of FC reagent was added with mixing. After 5 min, 300 μL of 20% (*w*/*v*) Na_2_CO_3_ solution was added. The reaction mixture was shaken correctly and then incubated for 2 h in the dark at room temperature. The absorbance of the mixture was determined at 765 nm using a spectrophotometer (Cary 50 probe, Varian, Belrose, NSW, Australia).

Quantification was carried out based on two standard curves prepared using gallic acid (y = 0.0456x − 0.0037, R^2^ = 0.9849) and quercetin (y = 0.0551x − 0.0067, R^2^ = 0.9823), and the results are expressed as milligrams of gallic acid (GAE) or quercetin (QE) per gram of leaf.

- Total flavonoid content

A colorimetric assay was used with some modifications [45]. An aliquot of the appropriately diluted infusion was vigorously mixed with 100 μL of 10% (*w*/*v*) AlCl_3_ solution. The absorbance of the mixture was determined at 440 nm using a spectrophotometer (Cary 50 probe, Varian, Australia).

The quantification was carried out by interpolation in a quercetin calibration curve (y = 0.1043x − 0.0395, R^2^ = 0.9959), and the results are expressed as milligrams of quercetin equivalent (QE) per gram of leaf.

- Ultra-High-Performance Liquid Chromatography Mass Spectrometry (UHPLC-MS) analysis

An Ultimate 3000 ultra-performance liquid chromatography (UPLC) system (Dionexcorp., Sunnyvale, CA, USA) with photodiode array detection (PAD) was coupled to a Bruker MicrOTOF-QII system by an electrospray ionization (ESI) interface (BrukerDaltonics, Billerica, MA, USA) for chromatographic and mass spectrometric (MS) analysis. A Hypersil C18 column (3.0 μm, 125 × 4.0 mm) (Varian) was used for chromatographic separation. The mobile phase consisted of 0.1% formic acid in water (A) and acetonitrile (B) using a gradient program of 5–35% (B) in 0–10 min, 35–80% (B) in 10–10.1 min, 80–80% (B) in 10.1–11, 80–45% (B) in 11–11.1, 45–5% (B) in 11.1–12 min, and 5% (B) in 12–15 min. The solvent flow rate was 0.5 mL/min, the column temperature was 30 °C, and the detection wavelength was 254 nm. The conditions of MS analysis in the negative ion mode were as follows: drying gas (nitrogen); flow rate, 8 L/min; gas temperature, 180 °C; scan range, 50–3000 *m*/*z*; end-plate offset voltage, −500 V; capillary voltage, 4500 V; nebulizer pressure, 2.5 bar.

The accurate mass data of the molecular ions were processed through DataAnalysis 4.0 software (BrukerDaltonics), which provided a list of possible elemental formulas using Generate Molecular Formula Editor, as well as a sophisticated comparison of the theoretical with the measured isotope pattern (σ value) for increased confidence in the suggested molecular formula [46]. The widely accepted accuracy threshold for confirmation of elemental compositions was established at 5 ppm. During the development of the UPLC method, external instrument calibration was performed using a 74900-00-05 Cole Palmer syringe pump (Billerica, MA, USA) directly connected to the interface with a sodium formate cluster solution. The calibration solution was injected at the beginning of each run, and all the spectra were calibrated before the compound identification.

### 4.8. Statistical Analysis

To perform statistical analyses, GraphPad Prism version 8.0.1 for Windows was used. Two-way repeated measures and one-way analysis of variance (ANOVA) tests were performed. The statistical test, Student–Newman–Keuls (S-N-K), was used for *post hoc* comparison. Significant differences were established at *p* values less than 0.05.

## 5. Conclusions

The LD_50_ of Gl leaves is greater than 5000 mg/kg in female rats and mice. The infusion of Gl leaves induces hypoglycemic and antihyperglycemic effects in vivo in normoglycemic and diabetic female rats at 250 and 500 mg/kg; the interaction between infusion (500 mg/kg) and metformin (300 mg/kg) results in hypoglycemic synergism. Gl leaves have potent antioxidant activity in vitro. These effects are attributed to alkaloids, phenols, and the identified compounds of chlorogenic acid, aucubin, rutin, and myricitrin in the plant leaves. The results of this research give scientific support to the traditional use of Gl leaves as an antidiabetic agent.

## Figures and Tables

**Table 1:** Identification of the main compounds in the aqueous extract obtained by the infusion of *G. laurifolia* leaves.

# Peak	Rt (min)	Name	[M-H]^−^_obs_	[M-H]^−^_exact_	Formula	Error	%RA
1	0.9	Unknown	533.1740	------	------	----	8.0
2	3.6	Aucubin	345.1176	345.1191	C_15_H_22_O_9_	−4.4	2.1
2	3.6	Benzyl 4-[2-(2-methoxyphenoxy)acetoxy]benzoate	391.1211	391.1187	C_23_H_20_O_6_	6.2	2.1
3	5.7	Ixoside	387.0895	387.0933	C_16_H_20_O_11_	5.4	9.5
4	5.8	[4-Methyl-7-(1-naphthylmethoxy)-2-oxo-2H-chromen-3-yl]acetic acid	373.1100	373.1081	C_23_H_18_O_5_	4.9	30.0
5	6.0	Chlorogenic acid	353.0837	353.0819	C_16_H_18_O_9_	−4.9	1.8
6–7	7.0–7.1	Chlorogenic acid isomer	353.0839	353.0819	C_16_H_18_O_9_	-4.6	25.0
8	7.7	Scopolin	353.0841	353.0878	C_16_H_18_O_9_	−6.3	0.8
9	7.8	7-[(4-Methoxybenzyl)oxy]-3-(4-methoxyphenyl)-2-methyl-4H-chromen-4-one	401.1411	401.1394	C_25_H_22_O_5_	−4.1	0.7
10	8.2	Clovin	755.2013	755.2040	C_33_H_40_O_20_	−3.6	1.3
11	8.8	Rutin	609.1448	609.1461	C_27_H_30_O_16_	−2.1	13.7
12	9.0	Myricitrin	463.0861	463.0882	C_21_H_20_O_12_	4.6	0.4
13	9.3	Luteolin 7-*O*-neohesperidoside	593.1487	593.1512	C_27_H_30_O_15_	4.2	2.0
14	10.0	Unknown	1127.3501	------	------		0.6
15	10.3	Unknown	1483.4842	------	------		2.2

Error [ppm]: the absolute value of the deviation between measured mass and theoretical mass of the selected peak in [ppm], [M-H]^−^_exact_: molecular weight exact, [M-H]^−^_obs_: molecular weight observed, Rt: retention time, %RA: % relative area; ----- unassigned.

## Data Availability

Data are contained within the article and Supplementary Materials.

## Supplementary Materials

The following supporting information can be downloaded at https://www.mdpi.com/article/10.3390/plants13223235/s1, Table S1: Medicinal plants orally consumed to treat DM in San Miguel Tecpan, Jilotzingo, State of Mexico, Mexico; Figure S1: Dissect analysis of the UHPLC-MS/MS chromatogram of infusion *G. laurifolia* leaves; Figure S2: Study area localization; Figure S3: Questionnaire of ethnobotanical study; Figure S4: Procedure used in the acute toxicity study to determinate the LD_50_ of leaves infusion of *G. laurifolia*; Figure S5: Procedure used in the pharmacological study to evaluate the effect of *G. laurifolia* in the streptozotocin-induced diabetes mellitus model.
