# Supplementary material for: Ethnopharmacological Study of Garrya laurifolia and Its Antidiabetic Effect in Rats
Source: Plants (Basel). 2024 Nov 18;13(22):3235. doi: 10.3390/plants13223235 (PMC11598156; doi:10.3390/plants13223235)
Supplement: Supplementary file 1 [file plants-13-03235-s001.zip › plants-3304822-supplementary.pdf]

plants

Supplementary Materials

Ethnopharmacological Study of *Garrya laurifolia* and Its Antidiabetic Effect in Rats

María Mirian Estévez-Carmona [1], Saudy Saret Pablo-Pérez [1,*,†], Jesús Eduardo Almanza-Cruz [1], María Estela Meléndez-Camargo [1,*,†], Daniel Arrieta-Baez [2], José Melesio Cristóbal-Luna [3] and Margarita Franco-Colín [4]

[1] Laboratorio de Farmacología y Toxicología Renal y Hepática, Departamento de Farmacia, Escuela Nacional de Ciencias Biológicas, Instituto Politécnico Nacional, Mexico City 07738, Mexico; mmestevez@ipn.mx (M.M.E.-C.); jalmancru@gmail.com (J.E.A.-C.)
[2] Centro de Nanociencias y Micro y Nanotecnologías, Instituto Politécnico Nacional, Mexico City 07738, Mexico; darrieta@ipn.mx
[3] Laboratorio de Toxicología Preclínica, Departamento de Fisiología, Escuela Nacional de Ciencias Biológicas, Instituto Politécnico Nacional, Mexico City 07738, Mexico; josmcl@hotmail.com
[4] Laboratorio de Metabolismo I, Departamento de Fisiología, Escuela Nacional de Ciencias Biológicas, Instituto Politécnico Nacional, Mexico City 07738, Mexico; mfrancoc@ipn.mx
* Correspondence: spablop@ipn.mx (S.S.P.-P.); emelendezc@hotmail.com (M.E.M.-C.)
† These authors equally contributed to the work.

Index

Title	No. page
**Table S1.** Medicinal plants orally consumed to treat DM in San Miguel Tecpan, Jilotzingo, State of Mexico, Mexico.	2
**Figure S1.** Dissect analysis of the UHPLC-MS/MS chromatogram of infusion *G. laurifolia* leaves.	3
**Figure S2.** Study area localization.	4
**Figure S3.** Questionnaire of ethnobotanical study.	5
**Figure S4.** Procedure used in the acute toxicity study to determinate the LD_{50} of leaves infusion of *G. laurifolia*.	6
**Figure S5.** Procedure used in the pharmacological study to evaluate the effect of *G. laurifolia* in the streptozotocin-induced diabetes mellitus model.	6

Table S1. Medicinal plants orally consumed to treat DM in San Miguel Tecpan, Jilotzingo, State of Mexico, Mexico.

No.	Plant specie	Family	Common name (s)	Method of obtaining	Plant part used	Quantity used	Preparation method	Frequency of use	Consumption, together with standard medication	Relative frequency of citation (RFC)	Demonstrated antidiabetic activity
1	*Opuntia ficus-indica*	Cactaceae	"Nopal"	Wild and home gardens collection, purchase	Raw spineless pulp pad	Half piece	Smoothie	Daily	Yes	0.80	Yes
2	*Psidium guajava*	Myrtaceae	Guava, "guayaba"	Wild collection	Leaves	Six leaves in a liter of water	Decoction or infusion	Daily	Yes	0.73	Yes
3	*Garrya laurifolia*	Garryaceae	Chichicaule, Bitter tree, "zapotillo"	Wild collection	Leaves	Five leaves in a liter of water	Infusion, direct chewing, decoction	Daily	Yes	0.68	No
4	*Silybum marianum*	Asteraceae	Cardo, "Cardo Mariano"	Wild collection, purchase	Flowers with stem and leaves	One piece in a cup of water	Decoction, infusion	Daily	Yes	0.57	Yes
5	*Eriobotrya japonica*	Rosaceae	"Níspero"	Wild collection	Leaves	Eight leaves in a liter of water	Infusion	Daily	Yes	0.23	Yes

The information was obtained through interviews and the questionnaire application during the ethnobotanical study in March 2023. RFC was obtained by calculating the number of informants who mentioned using the species by the number of informants participating in the survey.

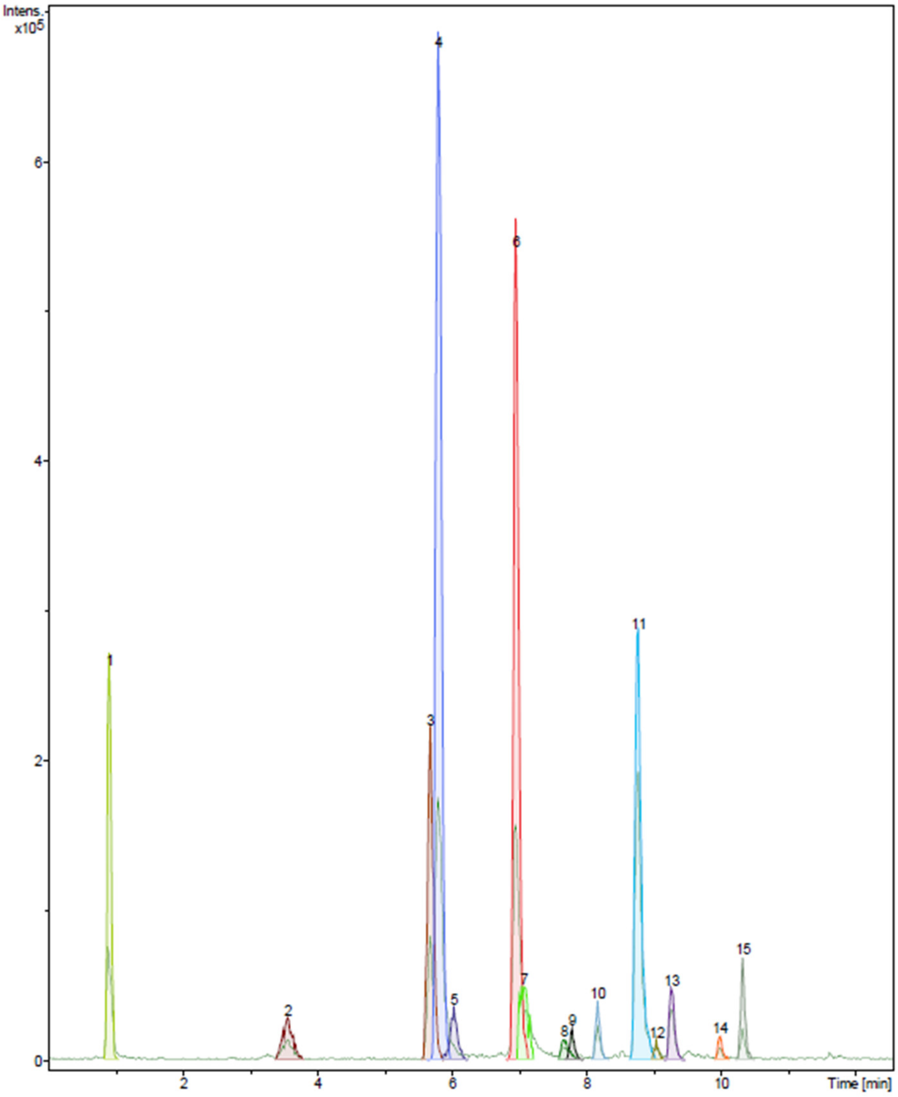

Figure S1. UHPLC-MS/MS chromatogram dissection analysis of infusion *G. laurifolia* leaves. 1: unknow; 2: aucubin and benzyl 4-[2-(2-methoxyphenoxy)acetoxy]benzoate; 3: ixoside; 4: [4-methyl-7-(1-naphthylmethoxy)-2-oxo-2H-chromen-3-yl]acetic acid; 5: chlorogenic acid; 6-7: chlorogenic acid isomer; 8: scopolin; 9: 7-[(4-methoxybenzyl)oxy]-3-(4-methoxyphenyl)-2-methyl-4H-chromen-4-one; 10: clovin; 11: rutin; 12: myricitrin; 13: luteolin 7-*O*-neohesperidoside; 14: unknown; 15: unknown.

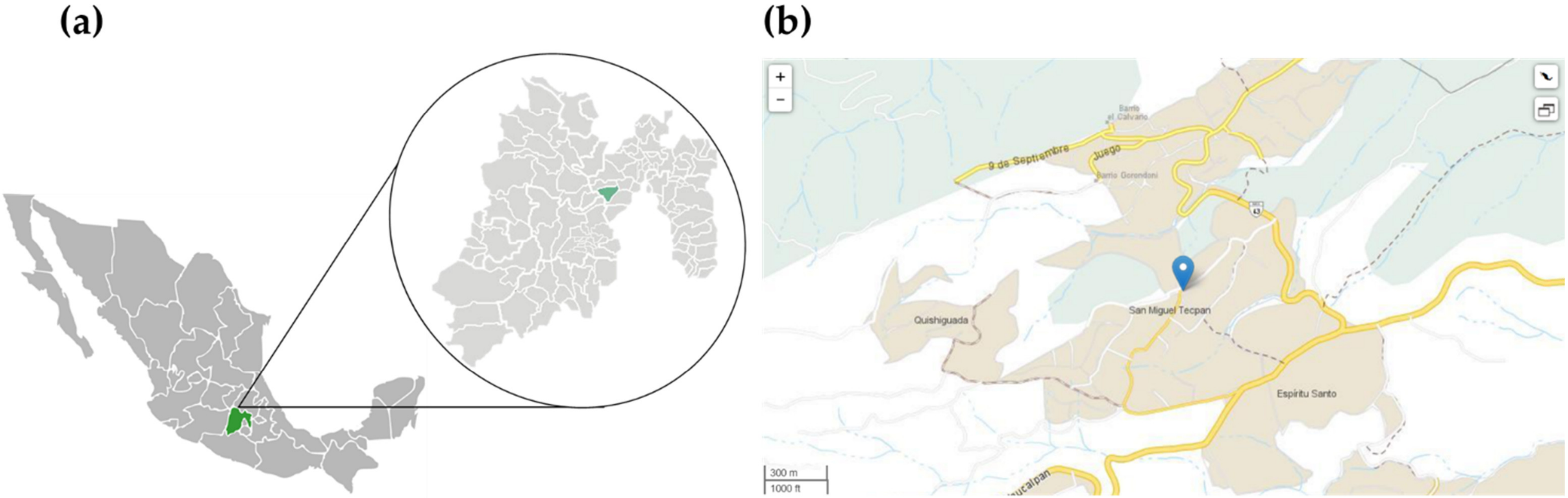

Figure S2. Study area localization. (**a**) Municipality of Jilotzingo, in the State of Mexico, Mexico. (**b**) San Miguel Tecpan community, topographic map with shading [29].

Ethnobotanical questionnaire of traditional use of medicinal plants

Questionnaire number: ___

1. Place and date of interview
2. Name or initials of the interviewed person
3. Age
4. Gender
5. Ethnic group
6. Level of studies
7. Occupation
8. Confirmation of visual identification of plant (s)
9. Common names of plant
10. Origin of traditional knowledge
11. Information about the environment, geography, and growth conditions of plant
12. Uses of plant

*If the last answer is associated with medicinal uses, continue with the following information

13. Illnesses treated with the vegetal species
14. If the medicinal plant is used exclusively or in combination with other plants or drugs
15. Parts and quantities of the plant consumed
16. Method of preparation of the traditional remedy
17. Frequency and duration of consumption of medicinal remedy
18. Additional information about the use of the plant or general observations

Figure S3. Questionnaire of ethnobotanical study.

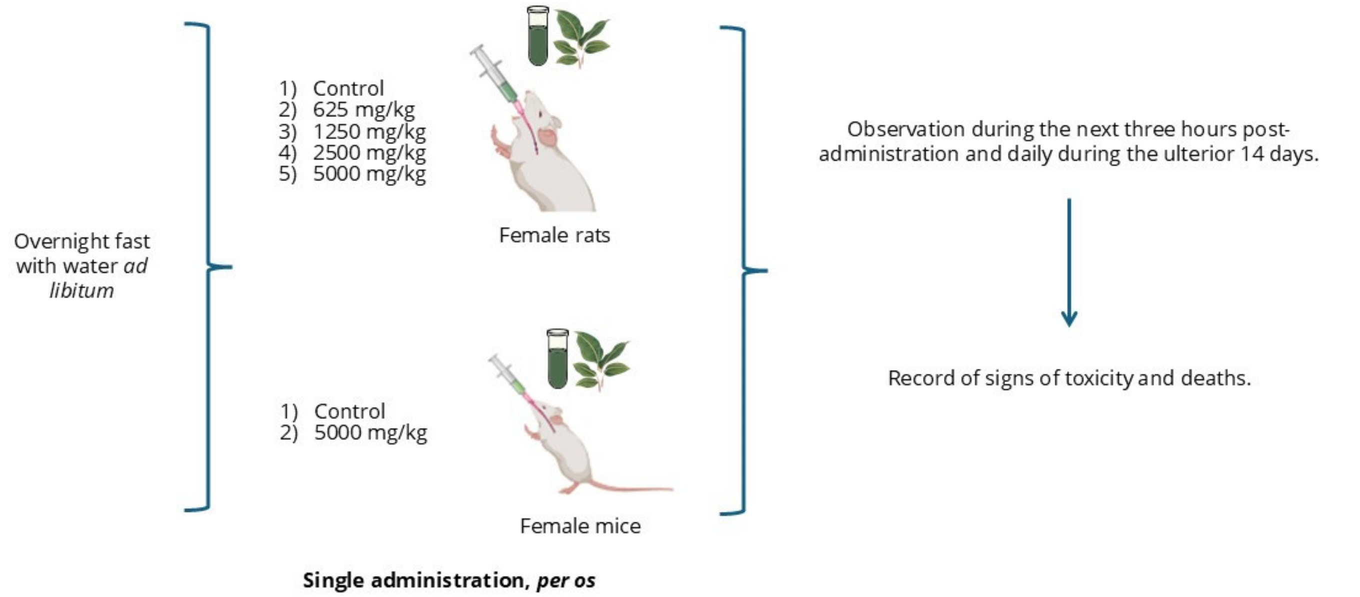

Figure S4. Procedure used in the acute toxicity study to determinate the LD_{50} of leaves infusion of *G. laurifolia*.

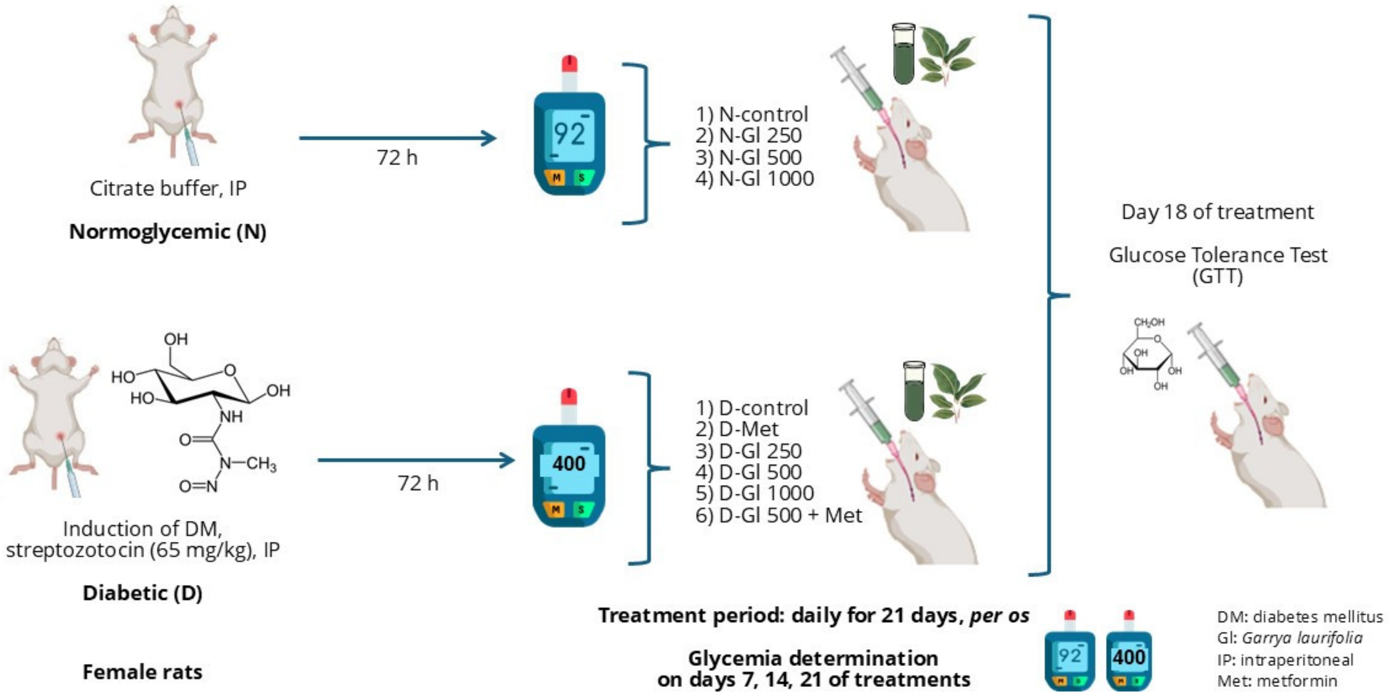

Figure S5. Procedure used in the pharmacological study to evaluate the effect of *G. laurifolia* in the streptozotocin-induced diabetes mellitus model.
